# mRNA vaccine against fibroblast activation protein ameliorates murine models of inflammatory arthritis

**DOI:** 10.2478/rir-2023-0013

**Published:** 2023-07-22

**Authors:** Xiaowei Zhang, Antony Jozic, Pingfang Song, Qiang Xu, Xiaofei Shi, Hong Wang, Lindsey Bishop, Hillary M Struthers, John Rutledge, Shuang Chen, Fei Xu, Meaghan H Hancock, Daocheng Zhu, Gaurav Sahay, Cong-Qiu Chu

**Affiliations:** Division of Arthritis and Rheumatic Diseases, Oregon Health & Science University, VA Portland Health Care System, Portland, Oregon 97239, USA; Department of Pharmaceutical Sciences, College of Pharmacy, Robertson Life Sciences Building, Oregon State University, Portland, Oregon 97201, USA; Department of Biomedical Engineering, Oregon Health & Science University, Portland, Oregon 97239, USA; Department of Ophthalmology, Casey Eye Institute, Oregon Health & Science University, Portland, Oregon 97239, USA; Department of Rheumatology, The First Hospital, Guangzhou University of Chinese Medicine, Guangzhou 51405, Guangdong Province, China; Department of Rheumatology, The First Hospital, Henan University of Science and Technology, Luoyang 471003, Henan Province, China; Department of Rheumatology, The Second Hospital, Wenzhou Medical University, Wenzhou 362000, Zhejiang Province, China; Vaccine and Gene Therapy Institute, Oregon Health & Science University, Beaverton, Oregon 97006, USA; Portland VA Research Foundation, Portland, Oregon 97239, USA; Department of Internal Medicine, Oregon Health & Science University, Portland, Oregon 97239, USA; Department of Hematology and Oncology, General Hospital of Ningxia Medical University, Yinchuan 750004, Ningxia Hui Autonomous Region, China; Shanghai Kexin Biotechnology, Co., Ltd., Shanghai, 201203, China

**Keywords:** rheumatoid arthritis, mRNA vaccine, fibroblasts, fibroblast activation protein

## Abstract

**Objective:**

Synovial fibroblasts in patients with rheumatoid arthritis (RA) contribute substantially to the perpetuation of synovitis and invasion to cartilage and bone, and are potential therapeutic targets. Fibroblast activation protein (FAP) is highly expressed by RA synovial fibroblasts and the expression is relatively specific. We tested whether FAP can serve as a molecular target to modulate synovial fibroblasts for therapy in experimental arthritis.

**Methods:**

mRNA encoding consensus FAP (cFAP) was encapsulated in lipid nanoparticles (LNP) and was injected intramuscularly as vaccine prior to induction of collagen-induced arthritis (CIA) and collagen antibody induced arthritis (CAIA) in mice. Development of CIA and CAIA was assessed clinically and by histology.

**Results:**

cFAP mRNA-LNP vaccine provoked immune response to cFAP and mouse FAP (mFAP); prevented onset of CIA in 40% of mice and significantly reduced the severity of arthritis. In CAIA, cFAP mRNA-LNP did not prevent onset of arthritis but significantly reduced the severity of arthritis.

**Conclusion:**

cFAP mRNA-LNP vaccine was able to provoke immune response to mFAP and suppress inflammatory arthritis.

## Introduction

A significant proportion of patients with rheumatoid arthritis (RA) fail to respond to current therapies although they are highly efficacious.^[1]^ Moreover, these therapeutics targeting immune cells or cytokines potentially compromise the immune system. Therefore, novel therapies with alternative targets are required.

RA is primarily an inflammation of the synovial joint. The synovium in RA grows enormously into a mass-like tissue that is largely contributed by fibroblasts which proliferate and are resistant to apoptosis.^[2,3]^ Fibroblasts build a stromal network which harbors immune and inflammatory cells. Moreover, fibroblasts actively interact with immune cells leading to persistent inflammation of the synovium with formation of new vessels and ectopic lymphoid follicles. In addition, RA fibroblasts can also produce inflammatory cytokines, participating in the inflammatory process. Thus, fibroblasts are a potential target for alternative therapy for RA.^[3, 4, 5]^ However, specifically targeting fibroblasts has been hampered due to the lack of a specific cell marker. Recent studies have revealed that fibroblast activation protein (FAP) may serve as a surrogate marker for activated RA fibroblasts. FAP is a type II transmembrane protein serving as a serine protease that cleaves the peptide bond between proline and other amino acids. FAP is exclusively expressed in fetal cells but not expressed in healthy adult tissue, except bone marrow derived mesenchymal stem cells and wounded tissues.^[6, 7, 8]^ In the RA synovium, FAP is highly expressed by fibroblasts in the lining and sub-lining layers.^[4,9]^ The expression is highly specific to RA fibroblasts since FAP is expressed in low levels in osteoarthritis and is not expressed in normal synovial fibroblasts. ^[9]^ Furthermore, depletion of FAP-expressing fibroblasts in serum transferred arthritis models reduced disease severity.^[4]^ Importantly, FAP gene knockout mice develop normally and have no clinical disease phenotype, suggesting that targeting FAP will be less likely to cause fatal adverse effects.^[10]^

FAP is highly expressed by fibroblasts in cancer stroma and by some cancer cells. Several strategies targeting FAP have been explored for cancer immunotherapy. These include vector-based, cell-based and DNA vaccines, monoclonal antibodies (mAb), and chimeric antigen receptor (CAR) T cells against FAP. However, phase I/II clinical trials with an anti-FAP mAb in cancer treatment did not show meaningful clinical efficacy probably because the unconjugated non-cytotoxic mAb failed to induce fibroblast cell death.^[11]^ However, DNA vaccines and CAR T cells against FAP have been demonstrated to be eficacious for immunotherapy in cancer models.^[12,13]^ Most recently, CAR T cells against FAP have shown to be highly effective to treat cardiac fibrotic pathologies and improve cardiac function without clinical toxicity in murine models.^[14,15]^ Here we report that an mRNA-based vaccine against FAP ameliorates mouse models of inflammatory arthritis.

## Methods

### Generation of cFAP mRNA and LNP Formulation

A consensus cDNA sequence for extracellular domains of FAP (cFAP) was generated by aligning cDNA sequences for mouse, rat and human FAP using software, MUSCLE version 3. The consensus cDNA sequence generated shared 95% similarity to mouse or human FAP cDNA sequences (see supplementary materials). A mutation, S624A was also introduced to inactivate the serine protease activity of the cFAP protein.^[12]^ mRNA for cFAP was custom transcribed and purified using the cFAP cDNA sequences by TriLink Biotechnologies. Enhanced green fluorescent protein (eGFP) mRNA was purchased from TriLink BioTechnologies Inc. (San Diego, CA, USA). mRNA was capped using TriLink CapClean co-transciptional capping method.

Lipid nanoparticles (LNP), consisting of Dlin-MC3-DMA, cholesterol, DMG-PEG2K, DSPC, and mRNA, were prepared using microfluidic mixing as described.^[16]^ mRNA was diluted in sterile 50 mmol/L citrate bufer, and lipid components were prepared in 100% ethanol at a 50:38.5:1.5:10 molar ratio. The lipid and mRNA solutions were mixed using the NanoAssemblr Benchtop (Precision Nanosystems, Inc., Vancouver, BC, Canada) at a 1:3 ratio, followed by overnight dialysis against sterile PBS using a Slide-A-Lyzer G2 cassette with 10,000 Da molecular-weight-cut-of (MWCO) (ThermoFisher Scientific Inc., Waltham, MA, USA). Dialyzed LNP solutions were concentrated using Amicon Ultra centrifugal filter units with a 10,000 Da MWCO (Millipore, Billerica, MA, USA). Hydrodynamic size and PDI of the LNP were measured in dynamic light scattering using the Zetasizer Nano ZSP (Malvern Instruments, Worcestershire, Malvern, UK). mRNA encapsulation was assayed using a modified Quant-iT RiboGreen RNA Assay kit (ThermoFisher Scientific, Waltham, MA, USA) and a multimode microplate reader (Tecan Trading AG, Männedorf, Switzerland). mRNA encapsulation was > 94%, hydrodynamic size of LNP was 91-96 nm and polydispersity index was 0.033-0.045.

### *In Vitro* Transfection of mRNA-LNP

For *in vitro* mRNA transfection for cFAP production, HEK293 cells were seeded on a 12-well plate at 3 × 10^5^ cells per well and allowed to attach overnight. Cells were treated with LNP encapsulating cFAP mRNA and harvested at 24 and 48 h. As a control, Lipofectamine™ 3000 Transfection Reagent (ThermoFisher Scientific, Waltham, MA, USA) was mixed with cFAP mRNA for transfection of cells. Supernatants were centrifuged at 500×*g* for 10 min at 4 °C. Cell-free media was supplemented with a protease and phosphatase inhibitor cocktail (ThermoFisher Scientific, Waltham, MA, USA) and used for capture ELISA and Western blotting. Transfected cells were lysed using RIPA bufer containing protease and phosphatase inhibitor cocktail (Bio-Rad, Hercules, CA, USA), followed by centrifugation at 16,000×*g* for 30 min at 4 °C. The cell lysate was collected for Western blotting. In some experiments, Golgi Plug (BioLegend, San Diego, CA, USA) was added in cultured cells for 5 h to stop protein secretion.

### Capture Enzyme-linked Immunosorbent Assay (ELISA) for Measuring cFAP

EIA plate was coated with 2 μg/mL of humanized anti-FAP monoclonal antibody (28H1, provided by Roche, Basel, Switzerland) in PBS overnight at 4 °C. Washed plate was blocked with 2% bovine serum albumin (BSA). Recombinant cFAP (custom produced by GenScript Biotech Corp. (Piscataway, NJ, USA) using the consensus FAP cDNA) was serial diluted at 1:2 in PBS containing 0.50% BSA and 0.05% Tween20 with top concentration of 2000 ng/mL to 2 ng/mL; and culture supernatant samples were incubated for 2 h at room temperature. After wash, rabbit anti-mouse FAP (R&D System, Minneapolis, MN, USA) was incubated at 2 μg/mL for 1 h at room temperature. Washed plate was incubated for 30 min at room temperature with goat anti-rabbit-horse radish peroxidase (HRP) diluted at 1:2000. After wash, substrate, 3,3’, 5,5’-Tetramethylbenzidine (TMB) was incubated for 30 min, and reaction was stopped with 2N sulfuric acid. Optical density (OD) was measured at 540 nm and concentration of cFAP in supernatant was derived against cFAP standard curve.

### Western Blotting for Detection of cFAP

Cell-free supernatants or cell lysates containing 30 μg of total protein were prepared in 1 × LDS sample buffer under reducing conditions, denatured at 70 °C for 10 min, and run on 4%-12% Bis-Tris gels or Tris-glycine gels, followed by dry transfer to PVDF membrane using iBlot 2 Dry Blotting System (ThermoFisher Scientific, Waltham, MA, USA). The blots were blocked using 5% BSA, 10% normal sheep serum and 5% skim milk for 1 h at room temperature (RT). The primary antibody, biotinylated sheep anti-FAP (Bio-Rad, Hercules, CA, USA) at 0.5 μg/mL was incubated for 1 h RT, then followed by streptavidin-HRP (1:1000, in blocking buffer). For detection, SuperSignal West Pico Plus Chemiluminescent Substrate and myECL imager (ThermoFisher Scientific, Waltham, MA, USA) were used.^[16]^

### Measurement of Antibodies to mFAP and cFAP

Mouse serum antibodies to mouse FAP (mFAP) and cFAP were determined by ELISA. EIA plate was coated with recombinant mFAP (R&D System, Minneapolis, MN, USA) or cFAP at 1 μg/mL in PBS at 4 °C overnight. Washed plate was blocked with 2% BSA. Mouse serum diluted at 1:10 in PBS containing 0.50% BSA and 0.05% Tween20 and incubated for 1 hour at room temperature. Mouse anti-mFAP (Santa Cruz, clone 73.30) and mouse anti-human FAP (cross reactive with cFAP, Santa Cruz, clone SS-13) was diluted at 1 μg/ mL and incubated to serve as positive controls for mFAP and cFAP respectively. After wash, biotinylated goat anti-mouse IgG (Jackson ImmunoResearch) diluted at 1:2000 was incubated for 1 h at room temperature. Washed plate was incubated with streptavidin-HRP at 1:1000 was incubated for 30 min at room temperature, after wash TMB was incubated for 30 min and reaction was stopped with 2N sulfuric acid. OD was measured at 540 nm. The antibody titer for mFAP and cFAP was expressed as arbitrary unit derived from division of the OD value of serum samples by OD value of positive control antibody respectively. Antibodies against eGFP were determined in a similar protocol using recombinant eGFP as antigen.

### Vaccination of cFAP and Induction of Arthritis

The animal experiments were approved by VA Portland Health Care System Institute Animal Care and Use Committee (#4593-20). Collagen-induced arthritis (CIA) and collagen antibody induced arthritis (CAIA) were induced in male DBA/1 mice (Jackson Laboratory, Bar Harbor, ME, USA) at 8-10 weeks. cFAP recombinant protein vaccination: 100 μg cFAP was emulsified in aluminum-based adjuvant and injected subcutaneously and 3 weeks post vaccination CIA was induced. Mouse cytomegalovirus (MCMV) vector vaccination: cDNA for cFAP was inserted into mouse cytomegalovirus (MCMV) BAC (Smith strain) between nucleotide 184293-184294 at IE2 locus by E/T recombination. The resulting MCMV-cFAP BAC contains the entire MCMV genome and the cFAP expression cassette. MCMV-cFAP was introduced by intra-peritoneal injection (2 x 10^6^ PFU/mouse). CIA was induced 3 weeks post MCMV-cFAP infection. cFAP mRNA-LNP vaccination: Mice were injected intramuscularly with cFAP mRNA-LNP at 1.5 μg/mouse at day 1 and a booster injection with the same dose at day 21. For CIA, at day 35, 100 μg chicken collagen type II (CII, Chondrex, Woodinville, WA, USA) emulsified in complete Freund’s adjuvant at the base of the tail. At day 56, 100 μg chicken CII was injected intraperitoneally (i.p.). For CAIA, at day 35, mice were injected i.p. with collagen antibody cocktail (Chondrex, Woodinville, WA, USA) at 5 mg/mouse, and followed by LPS at 25 μg/mouse at day 38. Severity of arthritis was monitored using a score system and by histology assessment as described.^[17]^

### Arthritis Clinical and Histological Scoring

Severity of mouse arthritis in CIA and CAIA were assessed using a scoring system as previously described.^[17]^

### Statistical Analysis

The incidence of arthritis between groups of mice was determined with the χ^2^ test. Clinical and histological severity of arthritis was analyzed with the non-parametric Mann-Whitney *U* test.

## Results

We first investigated the expression of FAP in two arthritis models: CIA in DBA/1 mice and zymosan-induced arthritis in SKG mice. FAP is highly expressed in the inflamed synovium of mice, in the lining layer and in the sub-lining layers. The pattern of expression is similar to that in RA synovium.^[9]^ Moreover, FAP can be detected in real-time in live animals with arthritis and its expression is positively correlated with the severity of arthritis (see Supplementary Figure S1). These results are consistent with previous reports where isotope labeled anti-FAP antibody was used to monitor arthritis severity by PET-CT scan.^[18,19]^ Importantly, FAP is not expressed in synovium of normal mice (see Supplementary Figure S1).

**Figure 1:**
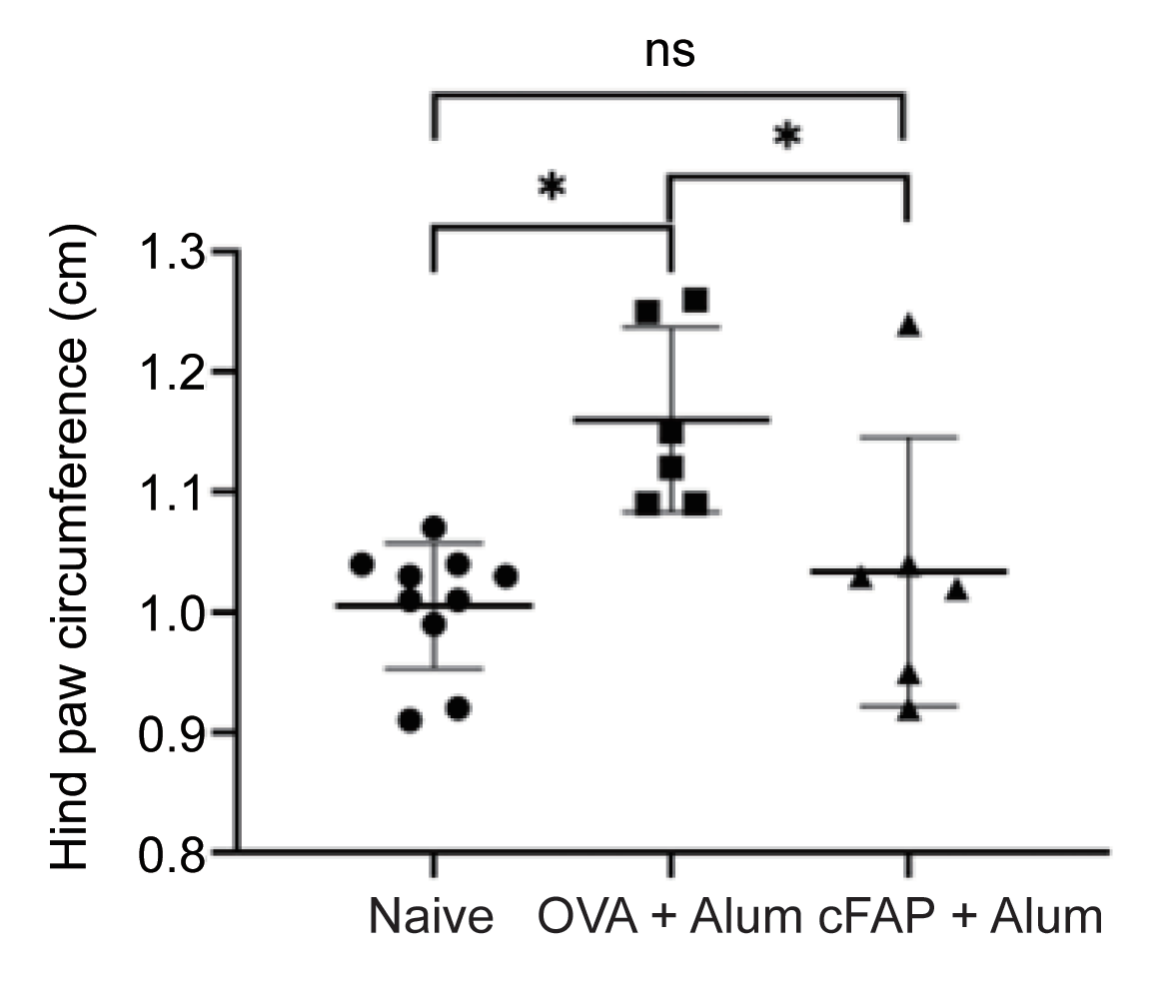
Suppression of arthritis by cFAP protein vaccination. cFAP or OVA at 100 μg was mixed with alum adjuvant and injected subcutaneously into male DBA/1 mice. Three weeks later, Collagen-induced arthritis (CIA) was induced and development of arthritis was monitored clinically. Alum, aluminum containing adjuvant; cFAP, consensus fibroblast activation protein; ns, not significant; OVA, ovalbumin; *P < 0.05.

**Figure 2:**
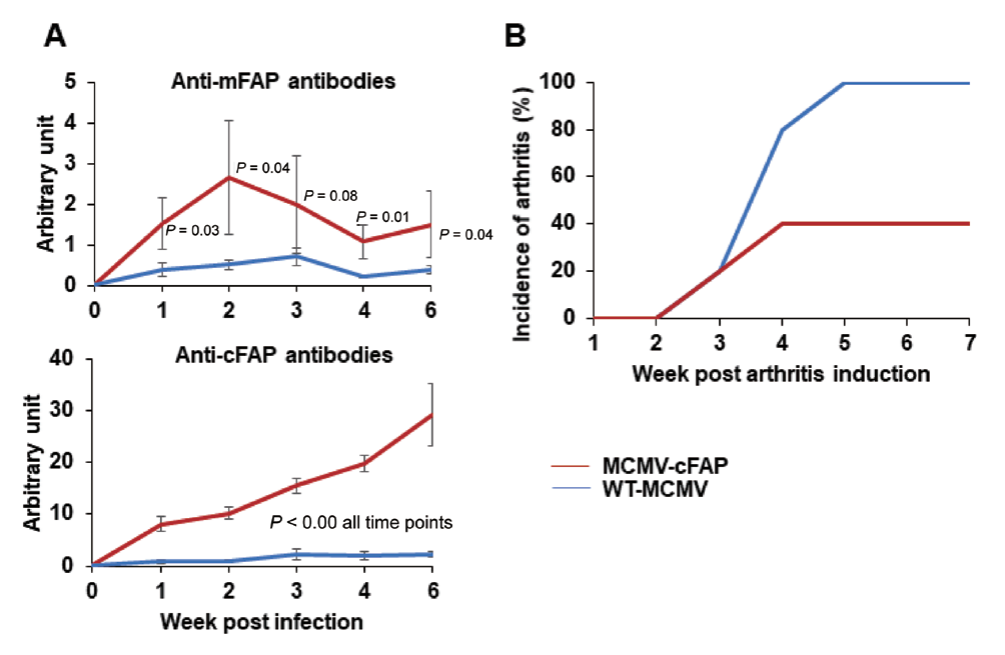
Mouse cytomegalovirus (MCMV) vectored cFAP induced anti-FAP antibody response *in vivo* and amelioration of arthritis. Male DBA/1 mice were infected with MCMV vectored cFAP DNA (MCMV-cFAP) or wild-type MCMV (WT-MCMV). (A) Serum anti-cFAP and mouse FAP (mFAP) were detected by ELISA. Antibody titer was expressed as arbitrary unit by ratio of optical density of serum over that of standard monoclonal anti-FAP antibodies. (B) CIA was induced 3 weeks after MCMV infection and development of arthritis was monitored clinically. cFAP, consensus fibroblast activation protein; CIA, collagen-induced arthritis.

We then tested whether an mRNA vaccine against FAP will have therapeutic effects on arthritis. In order to enhance the immunogenicity of a FAP mRNA vaccine, we designed a consensus cDNA sequence for the extracellular domains of FAP which shared 95% similarity to mouse and human FAP cDNA sequences (see Supplementary Figure S2). The effect of cFAP on reduction of arthritis was tested in CIA using two approaches. First, recombinant cFAP protein was expressed in CHO cells and purified, emulsified in aluminum-based adjuvant and injected subcutaneously (100 μg/mouse) in DBA/1 mice. Three weeks later, DBA/1 mice were induced for CIA. cFAP immunized group of mice (*n* = 10) had a reduced incidence of CIA to 20% *vs*. 80% in ovalbumin immunized group (*n* = 8) (Figure 1). Second, DBA/1 mice were infected with MCMV expressing cFAP (MCMV-cFAP) by intraperitoneal injection. Three weeks after MCMV-cFAP infection, mice developed antibodies to both cFAP and mFAP (Figure 2A). Further, MCMV-cFAP infected mice (*n* = 5) also showed a reduced incidence of CIA to 40% *vs*. 100% in MCMV vector only infected mice (*n* = 5) (Figure 2B). These results demonstrated that cFAP is immunogenic *in vivo*.

**Figure 3:**
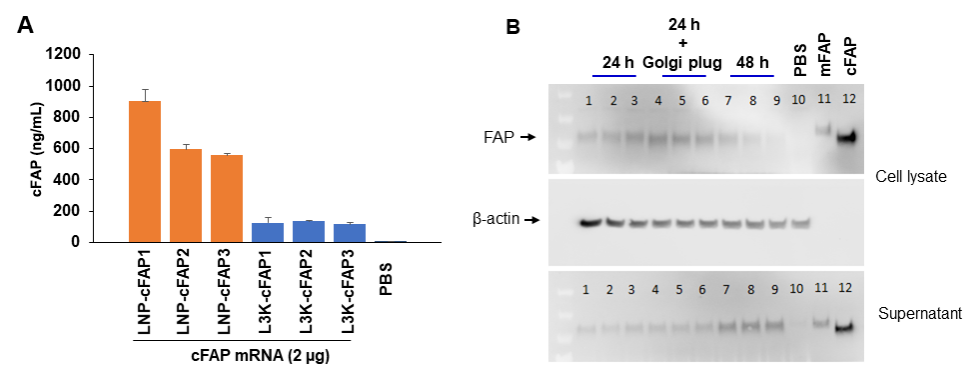
Expression of cFAP using mRNA-LNP transfection in vitro. HEK239 cells were incubated with cFAP mRNA-LNP or Lipofectamine™ 3000 Transfection Reagent (L3K) at 2 μg per 5 x 10^5^ cells. (A) Supernatants were harvested at 48 h post-transfection and cFAP were measured by a capture ELISA (showing 3 experiments and each condition was measured in triplicate). (B) In separate experiments, supernatants and cells were harvested at 24 and 48 h post-transfection for Western blotting. In some experiments, Golgi Plug was added for 5 h to block secretion of cFAP. mFAP, recombinant mouse FAP; cFAP, recombinant consensus FAP.

**Figure 4:**
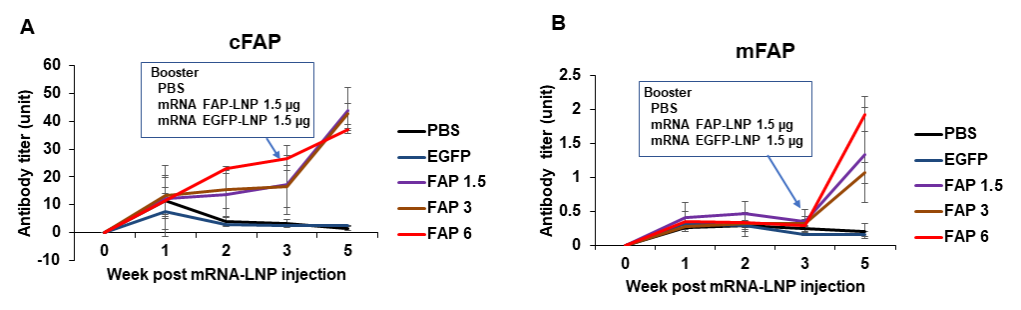
Antibody production in mice immunized with cFAP mRNA-LNP. Male DBA/1 mice were injected intramuscularly with cFAP mRNA-LNP in 3 dose groups (1.5, 3 or 6 μg/mouse), or eGFP mRNA-LNP eGFP (1.5 μg/mouse) at week 0; at week 3, a booster cFAP mRNA-LNP (all at 1.5 μg/mouse) or eGFP mRNA-LNP (1.5 μg/mouse) was injected. Serum was collected weekly for measurement of antibodies to cFAP (A) and mFAP (B) as in Figure 2 (n = 5 in each group). cFAP, consensus fibroblast activation protein; EGFP, enhanced green fluorescent protein; LNP, lipid nanoparticle; PBS, phosphate buffered saline.

**Figure 5:**
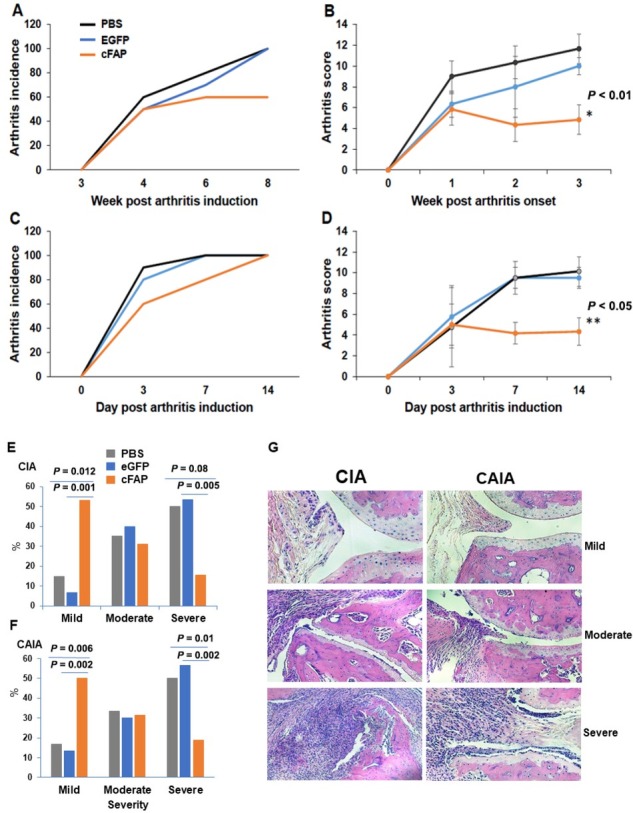
Suppression of arthritis by cFAP LNP-mRNA vaccine. Male DBA/1 mice were injected intramuscularly with cFAP mRNA-LNP (1.5 μg/mouse) or eGFP mRNA-LNP (1.5 μg/mouse) at week 0, then a booster injection was given in the same dose at week 3. Two weeks after the booster of cFAP LNP-mRNA, collagen-induced arthritis (CIA) or collagen antibody-induced arthritis (CAIA) was induced. Development of arthritis was assessed clinically (A-D) and by histology (E-G). CIA (A, B and E); CAIA (C, D, F). n = 6 in each group; *P < 0.01; **P < 0.05.

We next tested whether cFAP mRNA-LNP is immunogenic *in vivo*. cFAP mRNA-LNP was first tested and confirmed for expression of cFAP protein *in vitro* in HEK293 cells (Figure 3A, 3B) as detected by ELISA and western blotting. Mice with intramuscular injection of cFAP mRNA-LNP generated antibodies to cFAP and mouse FAP (mFAP), whereas those with eGFP mRNA-LNP injection generated antibodies to eGFP but not to cFAP or mFAP (Figure 4A, 4B). We then tested whether cFAP mRNA-LNP vaccine will be able to suppress arthritis. After two doses of cFAP mRNA-LNP vaccine (*n* = 10), 60% of mice developed CIA while 100% of mice in eGFP mRNA-LNP vaccinated (*n* = 8) or PBS treated (*n* = 8) groups developed CIA (Figure 5A); those animals that developed arthritis in cFAP mRNA-LNP treated group had a less severe course of the disease (Figure 5B, 5E, 5G). In the CAIA model, cFAP mRNA-LNP vaccine did not prevent arthritis (Figure 5C), but was able to reduce the severity of arthritis (Figure 5D, 5F, 5G).

## Discussion

LNP encapsulated mRNA vaccines have proven to be effective and safe against SARS-CoV-2 virus. Both LNP and mRNA components contribute to the immunogenicity of the vaccine.^[20]^ This rapid and scalable platform for vaccine development has broad applications beyond virus infections. In this study, we have shown that an mRNA-LNP based vaccine against FAP reduced the severity of experimental arthritis in mice. Vaccinated mice produced antibodies against mFAP, indicating that cFAP mRNA-LNP elicited an immune response to mFAP *in vivo*. FAP, expressed by activated fibroblasts, has been studied extensively as a target for ablation of fibroblasts in animal models of cancer immunotherapy,^[12,13]^ fibrotic pathologies,^[14,15]^ and in arthritis models.^[4,21]^ Moreover, studies using a ^68^Ga-labeled FAP inhibitor have shown *in vivo* tracer accumulation in arthritic joints in a newly diagnosed RA patient.^[22]^ *In vitro* experiments on explant RA synovium have also demonstrated that FAP-targeted photodynamic therapy selectively induces cell death of fibroblasts.^[22]^ These results suggest the feasibility of selectively ablation of FAP^+^ fibroblasts in RA using FAP as a target.

However, since FAP is an endogenous protein, concerns about toxicity or off-target effects upon depletion of FAP-expressing cells have been raised. Depletion of FAP^+^ stromal cells in muscle and bone marrow has been shown to cause muscle mass loss and cachexia in an adoptive transfer model of tumors.^[23]^ In contrast, data from several preclinical studies support the safety and viability of this approach. For example, mice with genetic ablation of FAP grow normally without pathological phenotypes.^[10]^ Many studies using DNA vaccines^[12]^ and CAR T cells against FAP reported successful inhibition of tumor growth^[13]^ and cardiac fibrosis^[14,15]^ without severe clinical toxicity or impaired wound healing. Since FAP is not expressed by normal fibroblasts, depletion of FAP-expressing cells does not affect normal fibroblasts. Data from the most recent study confirmed that depletion of FAP expressing fibroblasts in an arthritis model was not systemically toxic.^[4]^ In serum transfer arthritis model in transgenic FAP luciferase diphtheria toxin receptor reporter mice, FAP expressing cells were depleted by injection of diphtheria toxin after arthritis is established and arthritis was diminished, but no weight loss nor other signs of cachexia were observed.^[4]^ Additionally, no signs of toxicity were observed in our models (data not shown). Despite these observations, a careful safety study is required before this therapy can be translated for treating RA in humans.

Our study has limitations. It is not known how fibroblasts were manipulated *in vivo* after FAP mRNA-LNP vaccination, although the production of anti-mFAP antibodies indicates an immune response to mFAP. Whether cytotoxic T cells were generated and participate in the attack to fibroblasts was not explored. Further studies to delineate the *in vivo* immune responses to mFAP and effects on fibroblast function and survival are required. It is interesting and intriguing that FAP mRNA-LNP vaccination did not prevent onset of CAIA although it reduced arthritis severity. These findings may suggest that fibroblasts are actively involved in the priming phase of CIA where anti-collagen type II antibodies are produced in initiating arthritis.^[24]^ Whereas, in CAIA, which represent the inflammatory phase of CIA, the injected exogenous anti-collagen type II antibodies bypass the *in vivo* priming phase. Obviously, formal studies are required to verify these explanations.

Nevertheless, the mechanisms of action and target in this study are distinctly different from those of current biological therapeutic agents. Several strategies of targeting fibroblasts for treating RA have been explored in preclinical models of arthritis.^[5]^ In addition to inhibition of growth and promotion of death of fibroblasts, disrupting fibroblast interaction with extracellular matrix or endothelial cells also showed therapeutic effectiveness.^[25,26]^ However, targeting fibroblasts alone may not be a viable monotherapy for RA in clinical practice, but rather a complementary therapy to currently available modalities. Fibroblasts are not essential components of the immune system for host defense. Therefore, combining fibroblast targeted therapy with currently available biological therapeutic agents such as tumor necrosis factor inhibitors can be a viable therapeutic strategy to achieve a long-term remission with little or no more increased risk in causing serious adverse effects such as infections in patients with RA.

## Supplementary Material

Supplementary Materials
