# Supplementary material for: mRNA vaccine against fibroblast activation protein ameliorates murine models of inflammatory arthritis
Source: Rheumatol Immunol Res. 2023 Jul 22;4(2):90–7. doi: 10.2478/rir-2023-0013 (PMC10561064; doi:10.2478/rir-2023-0013)
Supplement: Supplementary file 1 — Supplementary Materials [file rir-2023-0013_supp.pdf]

Supplementary Materials

Supplementary Figure S1

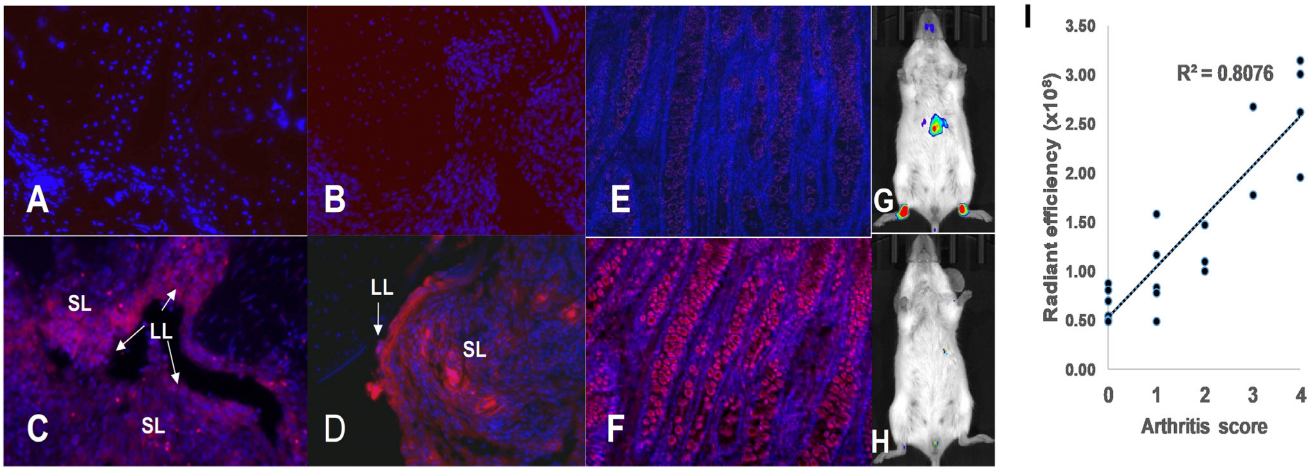

Figure S1: Expression of fibroblast activation protein in experimental arthritis. (A-F): Immunohistochemical staining of mouse joint with rat anti-mouse FAP (clone# 983802, R&D Systems) and followed by goat anti-rat IgG-NorthernLights NL557 (R&D Systems). (A) Normal mouse joint. (B) Arthritic joint of a mouse with collagen-induced arthritis (CIA) stained with an isotype control. (C) Arthritic joint of a mouse with CIA. (D) Arthritic joint from an SKG mouse with arthritis which was induced by intraperitoneal injection of zymosan. (E and F) Mouse colon cancer tissue stained with isotype control (E) and rat anti-mouse FAP (F). (G–I): FAP expression detected in live SKG mice with arthritis. (G) FAP in SKG arthritic joints detected with IRDye800CW labeled humanized monoclonal antibody to FAP (clone 28H1) by an in vivo image scanner, but not by normal human IgG (H). (I) FAP expression was positively correlated with severity of arthritis. LL, lining layer; SL, sub-lining layer.

Supplementary Figure S2

(a) Consensus cDNA sequences for fibroblast activation protein (cFAP)

2271 nucleotides (Kozak + IgE leader + consensus FAP extracellular domain)
GCCACCATGGACTGGACCTGGATCCTCTTCTTGGTGGCAGCAGCCACGCGAGTCCACTCC
TTACGTCCCTCAAGAGTTCACAACCCTGAAGGAAACACAAGGAGAGCTCTCACACTGAAGGATATTTTAAATGGAA-
CATTCTCATATAAAACATTTTTTCCAAACTGGATTTCAGGACAAGAATATCTTCATCAATCTGCAGATGATAACATAG-
TATTTTATAATATTGAAACAAGAGAATCATATATCATTTTGAGTAATAGCACCATGAAAAGTGTGAATGCTTCAGAT-
TATGGCTTATCACCTGATCGGCAATTTGTATATCTAGAAAGTGATTATTCAAAGCTTTGGAGATACTCATACACAG-
CAAATACTACATCTACGACCTTAGGAATGGGGAATTTGTAAGAGGATATGAGCTTCCTCGTCCAATTCAGTATCTAT-
GCTGGTCGCCTGTTGGGAGTAAATTAGCATATGTCTATCAAAACAATATTTATTTGAAACAAAGACCAGGAGATC-
CACCTTTTCAAATAACTTATACTGGAAGAGAAAATAAAATATTTAATGGAATCCCAGACTGGGTTTATGAAGAGGAAAT-
GCTTGCTACAAAATATGCTCTTTGGTGGTCTCCAAATGGAAAATTTTTGGCATATGCAGAATTTAATGATTCAGATATAC-
CAATTATTGCCTATTCCTATTATGGCGATGGACAGTATCCTAGAACAATAAATATTCCATACCCAAAGGCTGGAGCTA-
AGAATCCTGTTGTTCGTGTATTTATTGTTGACACCACCTACCCTCACTATGTGGGCCCCATGGAAGTGCCAGTTC-
CAGAAATGATAGCCTCAAGTGACTATTATTTCAGCTGGCTCACATGGGTGACTGATGAACGAGTATGTTTGCAGTG-
GCTAAAAAGAGTCCAGAATGTCTCGGTCCTGTCTATATGTGATTTCAGGGAAGACTGGCATACATGGGATTGTC-
CAAAGACCCAGGAGCATATAGAAGAAAGCAGAACTGGATGGGCTGGTGGATTCTTTGTTTCAACACCAGCTTTCAGC-
CATGATGCCATTTCATACTACAAAATATTTAGCGACAAGGATGGTTACAAACATATTCACTATATCAAAGACACTGTG-
GAAAATGCTATTCAAATTACAAGTGGCAAGTGGGAGGCCATATATATATTCAGAGTAACACAGGATTCACTGTTTTATTC-
TAGCAATGAATTTGAAGGTTACCCTGGAAGAAGAAACATCTACAGAATTAGCATTGGAAACTCTCCTCCGAGCAAGAAGT-
GTGTTACTTGCCATCTAAGGAAAGAAAGGTGCCAATATTACACAGCAAGTTTCAGCTACTACGCCAAGTACTATG-
CACTCGTCTGCTATGGCCCTGGCCTCCCCATTTCCACCCTTCATGATGGCCGCACAGACCAAGAAATACAAATATTG-
GAAGAAAACAAGGAATTGGAAAATGCTTTGAAAAATATCCAGCTGCCTAAAGAGGAAATTAAGAAGCTTGAAGAAGGT-
GAAATTACTTTATGGTACAAGATGATTCTTCCTCCTCAATTTGACAGATCAAAGAAGTACCCTTTGCTAATTCAAGTGTATG-
GTGGTCCCTGCAGCCAGAGTGTAAAGTCTGTATTTGCTGTTAATTGGATAACTTATCTTGCAAGTAAGGAGGGGATAGT-
CATTGCCTTGGTGGATGGTCGAGGCACTGCTTTCCAAGTGACAAATTCCTGTATGCAGTGTATCGAAAGCTGGGT-
GTTTATGAAGTTGAGGACCAGATCACAGCTGTCAGAAAATTCATAGAAATGGGTTTCATTGATGAAAAAAGAATAGC-
CATATGGGGCTGGGCCTACGGAGGATATGTTTCATCACTGGCCCTTGCATCTGGAACTGGTCTTTTCAAATGTGGAATAG-
CAGTGGCTCCAGTCTCCAGCTGGGAATATTACGCATCTATCTACACAGAGAGATTCATGGGCCTCCCAACAAAGGAT-
GATAATCTTGAGCACTATAAAAATTCAACTGTGATGGCAAGAGCAGAATATTTCAGAAATGTAGACTATCTTCTCATC-

CACGGAACAGCAGATGATAATGTGCACTTTCAGAACTCAGCACAGATTGCTAAAGCTTTGGTTAATGCACAAGTG-GATTTCCAGGCAATGTGGTACTCTGACCAGAACCATGGCATATCATCTGGCCGGTCCACGAACCACTTATATACCCACAT-GACCCACTTCCTCAAGCAATGCTTTTCTTTATCAGACTGA

(b)

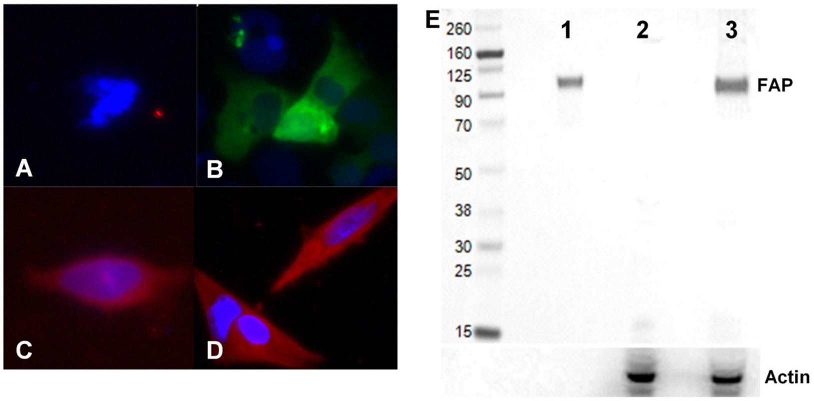

Figure S2: ***Consensus cDNA sequences for fibroblast activation protein (cFAP) and protein expression.*** *(a) A consensus cDNA sequence for extracellular domains was generated by aligning cDNA sequences for mouse, rat and human FAP using software, MUSCLE. The consensus cDNA sequence generated shared 95% similarity to mouse or human FAP cDNA sequences (see supplementary materials). A mutation, S624A was also introduced to inactivate the serine protease activity of the consensus FAP (cFAP) protein.[1] (b) Expression of cFAP. Plasmid DNA encoding cFAP was cloned into the modified pVax1 vector and transfected HEK293 cells with Lipofectamine 3000 reagents for 72 hours. (A) pVax1 vector only. (B) Green fluorescent protein (GFP)-DNA in pVax1. (C) Native mouse FAP-DNA in pVax1. (D) cFAP DNA in pVax1. (E) Western blot showing expression of consensus FAP protein. In the culture of transfected HEK293 cells, 3 μg/mL Brefeldin A was added and incubated for 5 hours to stop FAP protein being secreted before harvest of the cells. Cell lysate was probed with rabbit anti-mouse FAP and developed with goat anti-rabbit IgG-HRP and enhanced chemiluminescence. Lane 1: Recombinant muse FAP; Lane 2: pVax1 vector only transfected HEK293 cells; Lane 3: cFAP DNA in pVax1 transfected HEK293 cells.*

Reference

[1] Duperret EK, Trautz A, Ammons D, *et al*. Alteration of the Tumor Stroma Using a Consensus DNA Vaccine Targeting Fibroblast Activation Protein (FAP) Synergizes with Antitumor Vaccine Therapy in Mice. Clin Cancer Res. 2018;24:1190-1201.
